# Global Risk Assessment of the Occurrence of Bovine Lumpy Skin Disease: Based on an Ecological Niche Model

**DOI:** 10.1155/2023/2349173

**Published:** 2023-06-17

**Authors:** Qi An, Yue-peng Li, Zhuo Sun, Xiang Gao, Hong-bin Wang

**Affiliations:** ^1^College of Veterinary Medicine, Northeast Agricultural University, Harbin, China; ^2^Key Laboratory of the Provincial Education Department of Heilongjiang for Common Animal Disease Prevention and Treatment, College of Veterinary Medicine, Northeast Agricultural University, Harbin, China

## Abstract

Lumpy skin disease (LSD) is a highly contagious disease in bovine animals. An outbreak of LSD can cause devastating economic losses to the cattle industry. To investigate the distribution characteristics of historical LSD epidemics, LSD was divided into four phases for directional distribution analysis based on trends in epidemic prevalence. Ecological niche models were developed for LSD as well as for two vectors (*Stomoxys calcitrans* and *Aedes aegypti*), and global predictive maps were generated for the probability of LSD occurrence and the potential distribution of the two LSD vectors. The models had good predictive performance (the AUC values were 0.894 for the LSD model, 0.911 for the *S. calcitrans* model, and 0.950 for the *A. aegypti* model). The LSD combined vector prediction map was generated by combining the distribution maps of *Stomoxys calcitrans* and *Aedes aegypti*with fuzzy overlay tool in ArcGIS. The LSD combined vector prediction map was combined with the LSD prediction map to generate the LSD vector transmission risk map. The eastern and northwestern regions of North America, the eastern and northern regions of South America, the central and southern regions of Africa, the southern region of Europe, the northwestern and southeastern regions of Asia, and the eastern region of Australia were predicted to provide suitable environmental conditions for the occurrence of LSD. Cattle density, buffalo density, and bio2 (mean diurnal range) were identified as key variables for the occurrence of LSD. The findings of this study can be useful to policymakers in developing and implementing preventive measures of LSD for the health of cattle and the cattle industry.

## 1. Introduction

Bovine lumpy skin disease (LSD) is an infectious viral disease caused by the LSD virus (LSDV, genus goat pox virus; family Poxviridae) and is listed as a notifiable disease by the World Organization for Animal Health (WOAH) [1]. The LSDV shares antigenic similarities with sheep and goat pox viruses. However, it cannot be distinguished by routine serological testing [2]. The LSDV is highly host-specific and only infects and causes disease in bovids (mainly cattle and buffalo) under natural conditions, and no human infections have been reported [3]. The initial signs of infection in cattle include fever, depression, loss of appetite, excessive salivation, and markedly enlarged lymph nodes on the body surface [4]. Soon after the onset of fever, extensive skin nodules appear on the head, neck, limbs, udder, genitalia, and perineum of the affected cattle, gradually covering the entire body as the disease progresses. The skin nodules appear as firm, restricted, and rounded elevations, sometimes with lesions in the subcutaneous tissue and even in the muscle [5]. The morbidity and mortality in cattle caused by LSD are highly correlated with the strain of the virus, the breed of cattle, and immunization. Typically, the incidence of LSD ranges from 3–85%, but the mortality is relatively low (1–5%) [4]. LSD causes a reduction in the quality and quantity of milk production, damage to hides, weakness and weight loss, infertility, and abortions resulting in significant economic losses to the cattle industry [6].

LSD was first discovered in Zambia in 1929 and has shown endemicity mainly in Africa, including the Sahara and Madagascar [7]. Subsequently, the disease spread to most African countries, followed by the Middle East in 1986, and to southeastern and northeastern Europe since 2014, affecting the Caucasus countries, Kazakhstan, Russia, Greece, Armenia, Bulgaria, and the Republic of Macedonia [8]. The disease expanded to Asia since 2019, with outbreaks in India, Bangladesh, Nepal, Bhutan, Vietnam, and Myanmar [9]. As the epidemic spreads, the disease gradually threatens other European and Asian countries.

The primary source of infection for LSD is infected cattle. However, the transmission of the LSD virus from infected cattle is not fully understood. Direct contact with infected animals and transmission by blood-sucking insects (e.g., mosquitoes, flies, and ticks) have long been suggested as important routes for LSDV transmission [10]. Although direct contact is considered a less effective source of infection [11], studies assessing the risk of LSDV transmission by blood-sucking insects have shown that stable flies (*Stomoxys calcitrans*) and mosquitoes (*Aedes aegypti*) are the most likely vectors of LSD [12]. Indeed, the LSD-causing virus was detected in *S. calcitrans* caught on LSD-infected animals in the field [13]. *S. calcitrans* can also transmit LSDV from experimentally infected animals to naive cattle [14]. *Ae. aegypti* has been shown to transmit LSDV from infected to susceptible cattle and can be a more efficient vector than other mosquitoes [15, 16]. Therefore, this study considered two important vectors, *S. calcitrans* and *Ae. aegypti*, and explored their potential global distribution.

Spatiotemporal studies that focus on exploring the transmission and distribution characteristics of infectious diseases contribute to a better understanding of the patterns and risk factors of disease epidemics [17]. In addition, a historical spatiotemporal distribution of epidemics of infectious diseases may complement clinical and molecular studies of the diseases [18]. Therefore, spatial epidemiological tools are increasingly being applied to monitor and allocate resources in the face of emerging threats posed by infectious diseases to animals [19–21]. The ecological niche model (ENM) has widely been used to predict the potential distribution of disease and for disease risk mapping [22, 23]. The ENM explores the relationships between specific environmental variables and disease occurrence and helps to predict the potential distributions of a disease, which are important to respond to the spread and transmission of diseases [24]. In addition, predicting the spatial distribution of vectors by using ENM can inform decision-making to address control measures in targeted areas with the presence of vectors [25, 26]. In this study, a directional distribution analysis was performed using the standard deviation ellipse method to explore the historical spread of the LSD epidemic globally. A maximum entropy modeling was applied to LSD and LSD vector occurrence data to identify potential areas suitable for LSD amplification and vector transmission. The findings of this study will provide useful information for policymakers to develop tools and allocate resources for LSD pathogen detection and vector surveillance.

## 2. Materials and Methods

### 2.1. Occurrence Data Collection

Geographical coordinates of the historical LSD outbreak and LSD cases were obtained from the Food and Agriculture Organization of the United Nations (FAO) records (https://empres-i.apps.fao.org/). The records included 4316 global cases of LSD from 1/1/2006 to 18/09/2022. Occurrence records for *Ae. aegypti* and *S. calcitrans* from 2006 to 2022 were obtained from the Global Biodiversity Information Facility (GBIF) (https://www.gbif.org/) database. The GBIF database was searched for *Aedes aegypti* and *Stomoxys calcitrans* as keywords and clicked on “OCCURRENCES” in the results to display the distribution records of the species. The time period selected was 2006–2022 and then downloaded. After cleaning and sorting, only the occurrence records with specific latitude and longitude coordinates were retained and the rest were excluded from the analysis.

To reduce duplication and spatial autocorrelation in the data, the “trim duplicate occurrences” function in the ENMTool software was used to filter the occurrence records to ensure that there was only one record in a grid [27]. In the end, a total of 2196 occurrence records for LSD, 610 for *Ae. aegypti*, and 1485 for *S. calcitrans* were used to run the models (Table S1).

### 2.2. Directional Distribution Analysis

Directional distribution analysis is most often used in epidemiology to determine the directionality of disease distribution and transmission. A trend in the directional distribution of LSD was determined using a standard deviation ellipse, a method widely applied to study epidemics of infectious diseases in animals and humans. The standard deviation ellipse generates ellipsoidal polygons. The attributed values for these polygons include the *X* and *Y* coordinates of the mean center, long and short axes, and the orientation of the ellipse. Analysis of these features and elliptical attribution values helped us to determine whether the distribution of the LSD epidemic points was in a specific direction. Based on trends in the epidemic prevalence, we identified the epidemic progression patterns in four phases: 2006–2009, 2010–2013, 2014–2017, and 2018–September 2022. The standard deviation ellipse for each stage was calculated by the directional distribution tool within the spatial statistics tool of ArcGIS 10.2. The ellipse size was set to one standard deviation, incorporating approximately 68% of the number of cases in a phase.

### 2.3. Variable Collection

Environmental and topographic variables affect habitat conditions for arthropod survival and have been widely used in ecological niche modeling [28–32]. Environmental and topographic variables assembled for this study included 19 bioclimatic variables, solar radiation, wind speed, elevation, and normalized difference vegetation index (NDVI) (Table 1). Data for the 19 bioclimatic variables, solar radiation, wind speed, and elevation were downloaded from the WorldClim 2.1 database for 1970–2000. NDVI data were downloaded from the National Tibetan Plateau Data Center [33]. This dataset is the most recent version of the long series (1981–2015) normalized difference vegetation index product of the NOAA Global Inventory Monitoring and Modeling System (GIMMS). We used the maximum value compositing method to obtain the annual mean NDVI for 1981–2015 [34, 35]. The global density data for cattle and buffalo were obtained from the FAO livestock systems database [36, 37]. All raster data were at a resolution of 5 arc minutes. Before the model development, we performed multicollinearity and correlation analyses between 19 bioclimatic variables using the basic functions and the USDM package of *R* software. The variables with an absolute value of Pearson correlation coefficient >0.7 and a VIF value >5 were excluded from the model development. The final remaining bioclimatic variables were used with solar radiation, wind speed, altitude, and NDVI to construct the ecological niche model.

### 2.4. Ecological Niche Modeling

MaxEnt is a popular ecological niche modeling method with strong predictive performance [38, 39]. MaxEnt 3.4.1 software was employed to predict the current distribution of LSD, *Ae. aegypti*, and *S. calcitrans*. The model was set up as follows: 80% of the occurrence records are used to train the model and the remaining 20% are used to evaluate the model performance, the output format was logistic, the features were automatically selected, the regularization multiplier = 1, the maximum number of background points = 10000, the replicated run type was Bootstrap, and random seed was ticked. The model was repeated 10 times, and the average of the 10 results was taken as the final prediction. Model performance was assessed using the area under the receiver operating characteristic curve (AUC) criteria, with AUC values ranging from 0-1. The higher the AUC, the better the performance of the model. Furthermore, an AUC greater than 0.5 indicates that the predictive capacity of a model is higher than a model using random prediction.

### 2.5. LSD Transmission Risk Assessment

The model outputs were converted to raster files in ArcGIS 10.2 to produce an LSD occurrence suitability map and habitat suitability maps for *Ae. aegypti* and *S. calcitrans*. The fuzzy overlay tool in the spatial analysis tools of ArcGIS 10.2 allows for the analysis of the possibility that a phenomenon belongs to multiple ensembles during the multicriteria overlay analysis. The LSD combined vector prediction map was generated by combining *Ae. aegypti* and *S. calcitrans* habitat suitability maps with the fuzzy overlay tool. The overlay type “OR” was selected, meaning that each raster of the output layer was the maximum of the input layer. The LSD combined vector prediction map reflects the global distribution of LSD vectors. Then, the LSD combined vector prediction map was combined with the LSD occurrence suitability map by selecting “AND” as the overlay type in the fuzzy overlay tool. Selecting “AND” as the overlay type ensured that each raster of the output layer was the minimum of the input layer, kept the likelihood of LSD and the presence of the vector to a minimum, and produced a map that showed potential LSD vector transmission risk [40].

## 3. Results

### 3.1. Directional Distribution Analysis

**Figure 1:**
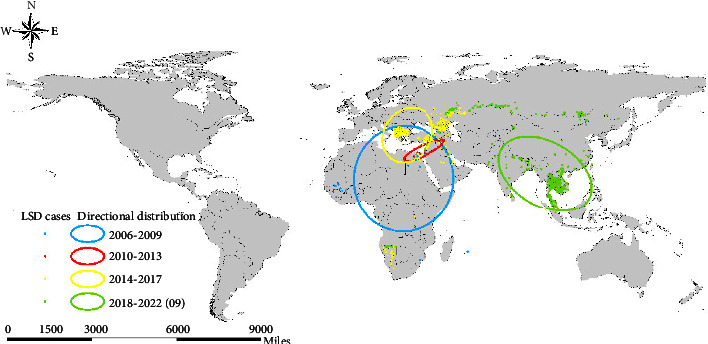
Directional distribution analysis of LSD cases in the world from 2006 to September 2022. The points and ellipses represent the LSD cases and standard deviation ellipses for the different phases. The blue color depicts 2006–2009, the red color depicts 2010–2013, the yellow color depicts 2014–2017, and the green color depicts 2018–September 2022.

The prevalence range of LSD from 2006 to September 2022 and the standard deviation ellipse for each stage are shown in Figure 1, and the attributed values of the standard deviation ellipse are shown in Table S2. During the phase 2006–2009, the LSD epidemic was most prevalent in the northern, western, and southern parts of the African region, with an insignificant directional trend. The long and short axes of the ellipse were comparable, and the center of the circle was located in western Sudan. In the phase 2010–2013, the LSD was prevalent in a small area within the western part of the Middle East. An immense flatness of the ellipse (the ratio of the difference between the long and short semiaxes to the long semiaxis) showed a significant directional distribution (northeast-southwest) of the LSD, with the center of the circle located in the sea near the western border of Israel. The LSD became a pandemic in southeastern Europe and the Middle East from 2014–2017. During the phase, the epidemic showed a northeast-southwest directional distribution with the center of the circle in western Turkey. In the last phase (2018 to September 2022), the LSD epidemic remained a pandemic in Southeast Asia, with an overall directional distribution trend (northwest-southeast) and the center of the ellipse in southeastern Myanmar.

### 3.2. Model Evaluation and Variable Contributions

The models were evaluated based on AUC values, and evaluation results are shown in Figure S1. The mean AUC values for the LSD, *S. calcitrans*, and *Ae. aegypti* models were 0.894, 0.911, and 0.950, respectively, demonstrating a strong performance of all three models. The variable sieving procedure was employed to select important variables contributing to each model. The results of selected variables and their contribution to the models are shown in Table 2. Variables that contributed more than 10% to a model were considered important. Cattle (cattle density), bio2 (mean diurnal range), and buffalo (buffalo density) had a contribution of more than 10% and were considered important variables in the LSD model. In the *Ae. aegypti* model, bio3 (isothermality), bio19 (mean precipitation of coldest quarter), srad (solar radiation), and NDVI were considered essential for the survival of *Ae. aegypti*. The important variables in the *S. calcitrans* model were bio19, bio6 (min temperature of the coldest month), and cattle.

### 3.3. Response Curves for Significant Variables in the Model

**Figure 2:**
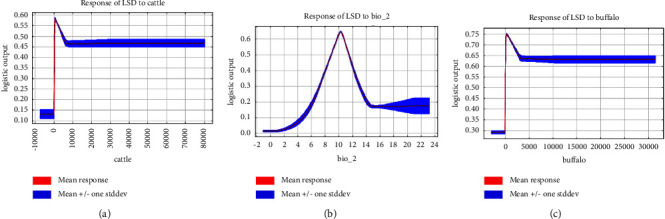
Response curves for important variables in the LSD model. The curves show the mean response of the 10 replicate Maxent runs (red) and the mean ± one standard deviation (blue): (a) cattle, (b) bio2, and (c) buffalo.

The response curves for the significant variables in the LSD model are shown in Figure 2. Although cattle and buffalo densities contributed more to the model, the probability of LSD occurrence remained relatively stable as both variables increased. The response curve for bio2 showed that the environmental suitability for LSD was higher at a mean diurnal range of approximately 7–13°C. The response curves for the LSD vectors are shown in Figures S2 and S3. *Ae. aegypti* occurred in areas with high levels of isothermality and solar radiation, appropriate winter precipitation, and suitable vegetation cover. Areas with mild winter temperatures, suitable precipitation, and the presence of cattle were suitable for *S. calcitrans*.

### 3.4. LSD and LSD Vectors Suitability Maps

**Figure 3:**
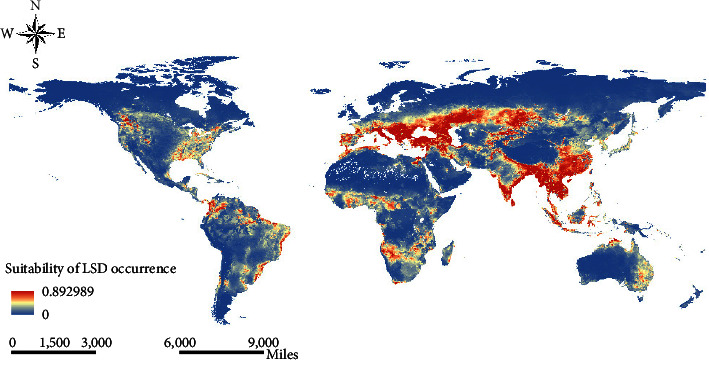
Suitability map for the LSD occurrence. The warmer colors depict areas of high suitability while cooler colors depict areas of low suitability.

The eastern and northwestern regions of North America, the eastern and northern regions of South America, the central and southern regions of Africa, the southern region of Europe, the northwestern and southeastern regions of Asia, and the eastern region of Australia were identified as suitable for the prevalence of LSD (Figure 3). The high-risk areas for LSD were concentrated in the southern part of Europe and the northwestern and southeastern parts of Asia. Southern North America, southeastern South America, southeastern Africa, southern Asia, and eastern Australia were identified as environmentally suitable for the subsistence of *Ae. aegypti* (Figure S4). The southern region of North America, the southeastern and northwestern regions of South America, the western region of Europe, the eastern region of Asia, and the eastern region of Australia may be the current ecological range of *S. calcitrans* (Figure S5).

### 3.5. Mapping LSD Transmission Risk Areas

**Figure 4:**
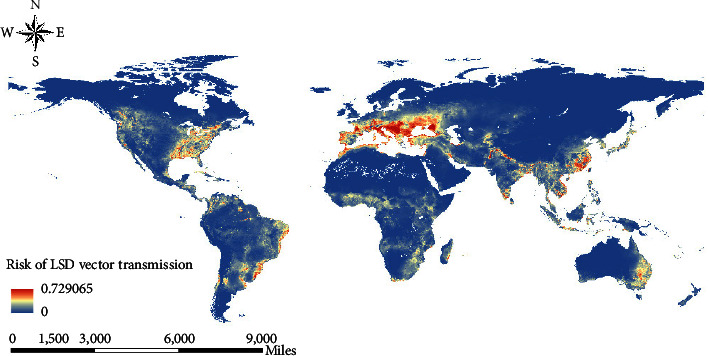
Risk map for LSD vector transmission. The warmer colors depict high-risk areas while cooler colors depict low-risk areas.

The LSD transmission risk map (Figure 4) was generated by combining the LSD occurrence suitability map (Figure 3) and the LSD combined vector prediction map (Figure S6). Results showed that the eastern region of North America, eastern South America, southern Europe, southern Asia, and eastern Australia might be at high risk of LSD transmission.

## 4. Discussion

The spread of the LSD epidemic is of international concern as it is an important statutory notifiable infectious disease. Despite the low mortality rate, LSD causes significant economic losses to the cattle industry. In addition, since LSD is considered a transboundary and trade band disease, it significantly impedes the international trade of livestock products. Using directional distribution analysis and ecological niche modeling, we explored the directional distribution of historical epidemics and areas of environmental suitability for the LSD globally. Due to logistical issues (e.g., delays in data reporting by the WOAH and FAO), September 2022 LSD outbreak data from India were not included in this study. Our results showed that the directional distribution trend in LSD from 2006–2009 was not apparent (Figure 1). However, there were clear northeast-southwest distribution patterns from 2010–2013 and 2014–2017. From 2018 to September 2022, the LSD epidemic had a northwest-southeast distributional trend. Over time, the directional shift in the LSD epidemic was reflected through a shift in the center of the standard deviation ellipse. LSD was first reported in Africa, then in the Middle East, where it became a small epidemic, then in southern Europe, where it showed a concentrated outbreak, and most recently in Asia, where it became a widespread epidemic. At present, the disease occurs in multiple continents, including Asia, Africa, and Europe. Throughout, the global occurrence pattern of LSD shows a clear cross-border spread. Long-distance migration of cattle and vector flight by wind contributes to the long-distance spread of LSD [41, 42]. Compared with other flying insects, *S. calcitrans* and *Ae. aegypti* may be effective vectors [12], and both may play important roles in cross-border transmission of LSD.

Our results showed that the high-risk areas for LSD were concentrated in the countries surrounding the Black Sea, north of the Mediterranean Sea, northwest and southeast Asia, and parts of Africa and the Americas (Figure 3). The environmental suitability for LSD disease across a large part of India reported in this study is supported by a severe LSD outbreak in India in September 2022 [43]. These findings also support the robustness and reliability of our modeling approach. The risk map suggested that the countries with no history of LSD or with a few cases, such as Colombia, Italy, Spain, France, Portugal, Slovenia, Slovakia, Hungary, Croatia, Bosnia and Herzegovina, Romania, Moldova, Georgia, Armenia, Azerbaijan, Tajikistan, Pakistan, China, and Myanmar, were at high-risk regions of LSD occurrence. These countries should be on alert for the importation and potential disease outbreak.

The response curves for the environmental variables showed that changes in each variable affect the probability of LSD incidence. The response curves for cattle density and buffalo density were generally smooth, with little effect of both variables on the incidence of LSD (Figure 2). However, the contribution of these variables to the model was reasonably high (cattle density: 44.2% and buffalo density: 10%). Previous mathematical modeling studies also reported similar results, with no clear relationships between cattle density and LSD infection rates [44]. The transmission patterns can be explained by indirect transmission [44]. This may be because direct animal contact is less rapid and efficient in transmitting the virus and does not play a significant role in transmission, with LSD more likely to be transmitted by blood-sucking insects [45, 46]. The response curve for bio2 showed that the temperature range of 7–13°C was suitable for LSDV. Lower latitudes experience greater daily variation in solar height and mean diurnal range, implying that the LSD may be more prevalent in the low and mid-latitudes with warm climatic conditions. The warmer and humid climates in lower latitudes can be conducive to the reproduction and survival of LSD vectors such as mosquitoes, flies, and ticks and transmitting LSD virus (ESM [4]).

One of the best ways to prevent and control infectious diseases is to cut off the transmission. The vectors of LSD are not well understood, but *Ae. aegypti* and *S. calcitrans* have been shown to play an important role in the transmission of LSD [12]. We collected historical records of the presence of *S. calcitrans* and *Ae. aegypti* and developed separate models using topography, climate, and vegetation to identify the distribution of LSD vectors and thus assess the risk of vector-borne LSD globally. Our results showed that appropriate temperatures and precipitation were necessary for *S. calcitrans* to survive the winter (Figure S3). *S. calcitrans* overwinter as eggs and larvae and are sensitive to changes in temperature and precipitation [47]. Excessive cold and dry conditions can prolong larval development and reduce the survival rate of the larvae [48]. Cattle density was also a significant predictor of *S. calcitrans* distribution, suggesting that cattle provide a blood meal for the survival of *S. calcitrans*. *Ae. aegypti* distribution was also influenced by temperature and precipitation in addition to vegetation cover. Results showed that *Ae. aegypti* performed well under high temperatures, sufficient moisture conditions, and enough vegetation cover (Figure S2). Temperature affects the survival, growth, and reproductive rate of *Ae. aegypti* [49, 50]. Precipitation and vegetation cover provide resting habitats and stimulate egg incubation [51, 52]. Our results also implied that the potential distribution ranges of *Ae. aegypti* and *S. calcitrans* overlapped little. We generated the LSD combined vector suitability map by overlaying the individual map of each species using the fuzzy overlay tool (Figure S6). A comparison of the LSD environmental suitability map with the LSD vectors suitability map suggested that most of the high-risk areas for LSD were also suitable for the survival of its vectors. The eastern United States, eastern and northwestern South America, Black Sea coast, northern Mediterranean countries, Pakistan, southern, eastern and northern of India, Bangladesh, Cambodia, Vietnam, southeastern China, and eastern Australia were considered at high risk of LSD vector transmission (Figure 4). In these countries and regions, the environmental suitability of LSD and the habitat suitability of the LSD vectors are both high and extremely suitable for the occurrence and spread of LSD. To reduce the risk of potential LSD outbreak and spread in these countries and regions, cattle should be vaccinated regularly, the movement of cattle should be controlled, vector surveillance should be increased, and regular disease surveillance should be conducted.

There are some limitations to our study. We did not have some relevant data to better predict the long-distance transmission of LSD. For instance, we could not consider the data related to long-distance cattle migration, which may be related to the cross-border spread of LSD [41]. Because of the paucity of information on LSD vectors and transmission mechanisms, we could not include several other potential insect vectors but only considered *S. calcitrans* and *Ae. aegypti*. Therefore, the LSD transmission risk map may be underestimated.

## 5. Conclusion

The directional distribution trend in the LSD epidemic was not obvious during the first phase (from 2006–2009). However, the LSD epidemic from 2010–2013 and 2014–2017 showed northeast-southwest distributional trends. From 2018 to September 2022, the disease showed a northwest-southeast distribution. Our model identified areas suitable for LSD occurrence and vector transmission. Cattle density, buffalo density, and bio2 (mean diurnal range) were the key factors influencing the occurrence of LSD. This study could be used for risk-based surveillance patterns that selectively target high-risk areas of LSD occurrence and vector transmission.

## Figures and Tables

**Table 1:** Variables used in the model.

	Variable name	Source
bio1	Annual mean temperature	WorldClim version 2.1
bio2	Mean diurnal range	WorldClim version 2.1
bio3	Isothermality (bio2/bio7) (×100)	WorldClim version 2.1
bio4	Temperature seasonality (standard deviation × 100)	WorldClim version 2.1
bio5	Max temperature of the warmest month	WorldClim version 2.1
bio6	Min temperature of the coldest month	WorldClim version 2.1
bio7	Temperature annual range (bio5-bio6)	WorldClim version 2.1
bio8	Mean temperature of the wettest quarter	WorldClim version 2.1
bio9	Mean temperature of the driest quarter	WorldClim version 2.1
bio10	Mean temperature of the warmest quarter	WorldClim version 2.1
bio11	Mean temperature of the coldest quarter	WorldClim version 2.1
bio12	Annual precipitation	WorldClim version 2.1
bio13	Precipitation of the wettest month	WorldClim version 2.1
bio14	Mean precipitation of the driest month	WorldClim version 2.1
bio15	Precipitation seasonality	WorldClim version 2.1
bio16	Precipitation of the wettest quarter	WorldClim version 2.1
bio17	Precipitation of the driest quarter	WorldClim version 2.1
bio18	Mean precipitation of the warmest quarter	WorldClim version 2.1
bio19	Mean precipitation of the coldest quarter	WorldClim version 2.1
srad	Solar radiation	WorldClim version 2.1
wind	Wind speed	WorldClim version 2.1
elev	Elevation	WorldClim version 2.1
cattle	Cattle density	FAO livestock systems
buffalo	Buffalo density	FAO livestock systems
ndvi	Normalized Difference Vegetation Index	National Tibetan Plateau Data Center

**Table 2:** Variables used in final models and variable contribution.

Variable name	Variable contribution (%)		
	LSD	*Aedes aegypti*	*Stomoxys calcitrans*
bio1			
bio2	10.5	0.8	
bio3	5.1	25.2	3.3
bio4			
bio5	2	0.4	1.9
bio6			23.2
bio7			
bio8		8.9	0.6
bio9	0.9	5.3	
bio10			
bio11			
bio12			
bio13			
bio14			
bio15	1.5	0.9	4.4
bio16			
bio17			
bio18	4.3	3.8	2.2
bio19	0.8	18	45.1
srad	7.9	16.6	0.6
wind	7.9	1.1	2.9
elev	2.3	0.7	2.6
cattle	44.2	1.3	11
buffalo	10	2.3	1
ndvi	2.5	14.8	1.1

## Data Availability

The datasets generated during and/or analyzed during the current study are available from the corresponding author upon reasonable request.

## Supplementary Materials

Supplementary Materials Figure S1: Results of the model evaluation based on the area under the receiver operating characteristic curve. The curves show the mean ROC of the 10 replicate Maxent runs (red) and the mean ± one standard deviation (blue). The black line indicates random prediction. (a) LSD model. (b) *Stomoxys calcitrans* model. (c) *Aedes aegypti* model. Figure S2: Response curves for important variables in the *Aedes aegypti* model. The curves show the mean response of the 10 replicate Maxent runs (red) and the mean ± one standard deviation (blue). (a) bio3. (b) bio19. (c) srad. (d) ndvi. Figure S3: Response curves for important variables in the *Stomoxys calcitrans* model. The curves show the mean response of the 10 replicate Maxent runs (red) and the mean ± one standard deviation (blue). (a) bio19. (b) bio6. (c) cattle. Figure S4. Habitat suitability map for *Aedes aegypti*. The warmer colors depict areas of high habitat suitability while cooler colors depict areas of low habitat suitability. Figure S5: Habitat suitability map for *Stomoxys calcitrans*. The warmer colors depict areas of high habitat suitability while cooler colors depict areas of low habitat suitability. Figure S6: Habitat suitability of LSD combined vector. The warmer colors depict areas of high habitat suitability while cooler colors depict areas of low habitat suitability. Table S1: Filtering of occurrence records. Table S2: Attributed values for the global LSD cases standard deviation ellipse, 2006–September 2022.

## References

[1] World Organisation for Animal Health (2022). Manual of diagnostic tests and vaccines for terrestrial animals 2022-lumpy skin diseases. *WOAH Terrestrial Manual*.

[2] Zhang M., Sun Y., Liu W., Liu R., Wang X., Bu Z. (2020). Isolation and identification of lumpy skin disease virus from the first outbreak in China. *Chinese Journal of Preventive Veterinary Medicine*.

[3] Kreindel S., Masiulis M., Skrypnyk A., Zdravkova A., Escher M., Raizman E. (2016). Emergence of lumpy skin disease in Asia and Europe. *Food and Agriculture Organization*.

[4] Tuppurainen E., Oura C. (2012). Review: lumpy skin disease: an emerging threat to Europe, the Middle East and Asia: emerging lumpy skin disease. *Transboundary and emerging diseases*.

[5] Kononov A., Prutnikov P., Shumilova I. (2019). Determination of lumpy skin disease virus in bovine meat and offal products following experimental infection. *Transboundary and emerging diseases*.

[6] Babiuk S., Bowden T., Boyle D., Wallace D. B., Kitching R. (2008). Capripoxviruses: an emerging worldwide threat to sheep, goats and cattle. *Transboundary and emerging diseases*.

[7] Wainwright S., El Idrissi A., Mattioli R., Tibbo M., Njeumi F., Raizman E. (2013). Emergence of lumpy skin disease in the Eastern Mediterranean Basin countries. *FAO Empres Watch*.

[8] jia W., Zhang L., He Y., Chi S., Wang X. (2021). Research progress on lumpy skin disease in cattles. *Animal Husbandry and Veterinary Medicine*.

[9] He X., Jing W., Fang Y. (2021). New epidemic trends of bovine lumpy skin disease and coping strategies in China. *Chinese Veterinary Science*.

[10] Annandale C. H., Holm D. E., Ebersohn K., Venter E. H. (2014). Seminal transmission of lumpy skin disease virus in heifers. *Transboundary and emerging diseases*.

[11] Tuppurainen E., Alexandrov T., Beltrán‐Alcrudo D. (2017). Lumpy skin disease field manual–A manual for veterinarians. *FAO Animal Production and Health Manual No*.

[12] Gubbins S. (2019). Using the basic reproduction number to assess the risk of transmission of lumpy skin disease virus by biting insects. *Transboundary and emerging diseases*.

[13] Kahana‐Sutin E., Klement E., Lensky I., Gottlieb Y. (2017). High relative abundance of the stable fly Stomoxys calcitrans is associated with lumpy skin disease outbreaks in Israeli dairy farms. *Medical and Veterinary Entomology*.

[14] Sohier C., Haegeman A., Mostin L. (2019). Experimental evidence of mechanical lumpy skin disease virus transmission by Stomoxys calcitrans biting flies and Haematopota spp. horseflies. *Scientific Reports*.

[15] Chihota C., Rennie L., Kitching R., Mellor P. (2001). Mechanical transmission of lumpy skin disease virus by *Aedes aegypti* (Diptera: Culicidae). *Epidemiology and Infection*.

[16] Chihota C., Rennie L., Kitching R., Mellor P. (2003). Attempted mechanical transmission of lumpy skin disease virus by biting insects. *Medical and Veterinary Entomology*.

[17] Sinha G., Mark D. M. (2005). Measuring similarity between geospatial lifelines in studies of environmental health. *Journal of Geographical Systems*.

[18] Qiu J., Li R., Xu X., Hong X., Xia X., Yu C. (2014). Spatiotemporal pattern and risk factors of the reported novel avian-origin influenza A (H7N9) cases in China. *Preventive Veterinary Medicine*.

[19] Gao H., Ma J. (2021). Spatial distribution and risk areas of foot and mouth disease in mainland China. *Preventive Veterinary Medicine*.

[20] Ma J., Xiao J., Gao X., Liu B., Chen H., Wang H. (2017). Spatial pattern of foot-and-mouth disease in animals in China, 2010–2016. *PeerJ*.

[21] Rahman A. K. M. A., Islam S. S., Sufian M. A., Talukder M. H., Ward M. P., Martínez-López B. (2020). Peste des Petits Ruminants risk factors and space-time clusters in Bangladesh. *Frontiers in Veterinary Science*.

[22] Dicko A. H., Lancelot R., Seck M. T. (2014). Using species distribution models to optimize vector control in the framework of the tsetse eradication campaign in Senegal. *Proceedings of the National Academy of Sciences*.

[23] Escobar L. E. (2020). Ecological niche modeling: an introduction for veterinarians and epidemiologists. *Frontiers in Veterinary Science*.

[24] Warren D. L., Seifert S. N. (2011). Ecological niche modeling in Maxent: the importance of model complexity and the performance of model selection criteria. *Ecological Applications*.

[25] Mweya C. N., Kimera S. I., Kija J. B., Mboera L. E. (2013). Predicting distribution of *Aedes aegypti* and *Culex pipiens* complex, potential vectors of Rift Valley fever virus in relation to disease epidemics in East Africa. *Infection Ecology and Epidemiology*.

[26] Ren Z., Wang D., Ma A. (2016). Predicting malaria vector distribution under climate change scenarios in China: challenges for malaria elimination. *Scientific Reports*.

[27] Warren D. L., Glor R. E., Turelli M. (2010). ENMTools: a toolbox for comparative studies of environmental niche models. *Ecography*.

[28] Clarke-Crespo E., Moreno-Arzate C. N., López-González C. A. (2020). Ecological niche models of four hard tick genera (Ixodidae) in Mexico. *Animals*.

[29] Leta S., Fetene E., Mulatu T. (2019). Modeling the global distribution of Culicoides imicola: an Ensemble approach. *Scientific Reports*.

[30] Liu B., Gao X., Ma J., Jiao Z., Xiao J., Wang H. (2018). Influence of host and environmental factors on the distribution of the Japanese encephalitis vector Culex tritaeniorhynchus in China. *International Journal of Environmental Research and Public Health*.

[31] Liu B., Ma J., Jiao Z., Gao X., Xiao J., Wang H. (2021). Risk assessment for the Rift Valley fever occurrence in China: special concern in south‐west border areas. *Transboundary and emerging diseases*.

[32] Nnko H. J., Gwakisa P. S., Ngonyoka A., Sindato C., Estes A. B. (2021). Potential impacts of climate change on geographical distribution of three primary vectors of African Trypanosomiasis in Tanzania’s Maasai Steppe: G. m. morsitans, G. pallidipes and G. swynnertoni. *PLoS Neglected Tropical Diseases*.

[33] The National Tibetan Plateau Data Center (2018). *Global GIMMS NDVI3g V1 Dataset (1981-2015)*.

[34] Holben B. N. (1986). Characteristics of maximum-value composite images from temporal AVHRR data. *International Journal of Remote Sensing*.

[35] Julien Y., Sobrino J. A. (2019). Optimizing and comparing gap-filling techniques using simulated NDVI time series from remotely sensed global data. *International Journal of Applied Earth Observation and Geoinformation*.

[36] Gilbert M., Nicolas G., Cinardi G. (2018). Global buffaloes distribution in 2010. *Scientific Data*.

[37] Gilbert M., Nicolas G., Cinardi G. (2018). Global cattle distribution in 2010. *Scientific Data*.

[38] Elith J., Graham C., Anderson R. (2006). Novel methods improve prediction of species’ distributions from occurrence data. *Ecography*.

[39] Phillips S. J., Dudík M. (2008). Modeling of species distributions with Maxent: new extensions and a comprehensive evaluation. *Ecography*.

[40] Fekede R. J., Gils H., Huang L., Wang X. (2019). High probability areas for ASF infection in China along the Russian and Korean borders. *Transboundary and emerging diseases*.

[41] Ince O. B., Çakir S., Dereli M. A. (2016). Risk analysis of lumpy skin disease in Turkey. *Indian Journal of Animal Research*.

[42] Klausner Z., Fattal E., Klement E. (2017). Using synoptic systems’ typical wind trajectories for the analysis of potential atmospheric long‐distance dispersal of lumpy skin disease virus. *Transboundary and emerging diseases*.

[43] Kumar N., Tripathi B. N. (2022). *A Serious Skin Virus Epidemic Sweeping through the Indian Subcontinent Is a Threat to the Livelihood of Farmers*.

[44] Magori-Cohen R., Louzoun Y., Herziger Y. (2012). Mathematical modelling and evaluation of the different routes of transmission of lumpy skin disease virus. *Veterinary Research*.

[45] Lubinga J., Tuppurainen E., Stoltsz W., Ebersohn K., Coetzer J., Venter E. (2013). Detection of lumpy skin disease virus in saliva of ticks fed on lumpy skin disease virus-infected cattle. *Experimental & Applied Acarology*.

[46] Tuppurainen E., Lubinga J. C., Stoltsz W. H. (2013). Mechanical transmission of lumpy skin disease virus by Rhipicephalus appendiculatus male ticks. *Epidemiology and Infection*.

[47] Phasuk J., Prabaripai A., Chareonviriyaphap T. (2013). Seasonality and daily flight activity of stable flies (Diptera: muscidae) on dairy farms in Saraburi Province, Thailand. *Parasite*.

[48] Friesen K., Berkebile D., Wienhold B., Durso L., Zhu J., Taylor D. B. (2016). Environmental parameters associated with stable fly (Diptera: muscidae) development at hay feeding sites. *Environmental Entomology*.

[49] Focks D. A., Haile D., Daniels E., Mount G. A. (1993). Dynamic life table model for *Aedes aegypti* (Diptera: Culicidae): analysis of the literature and model development. *Journal of Medical Entomology*.

[50] Simoy M. I., Simoy M. V., Canziani G. A. (2015). The effect of temperature on the population dynamics of *Aedes aegypti*. *Ecological Modelling*.

[51] Allan S. A., Kline D. L., Walker T. (2009). Environmental factors affecting efficacy of bifenthrin-treated vegetation for mosquito control. *Journal of the American Mosquito Control Association*.

[52] Lega J., Brown H. E., Barrera R. (2017). *Aedes aegypti* (Diptera: Culicidae) abundance model improved with relative humidity and precipitation-driven egg hatching. *Journal of Medical Entomology*.

