# Supplementary material for: Global Risk Assessment of the Occurrence of Bovine Lumpy Skin Disease: Based on an Ecological Niche Model
Source: Transbound Emerg Dis. 2023 Jun 17;2023:2349173. doi: 10.1155/2023/2349173 (PMC12016810; doi:10.1155/2023/2349173)
Supplement: Supplementary Materials — Figure S1: Results of the model evaluation based on the area under the receiver operating characteristic curve. The curves show the mean ROC of the 10 replicate Maxent runs (red) and the mean ± one standard deviation (blue). The black line indicates random prediction. (a) LSD model. (b) Stomoxys calcitrans model. (c) Aedes aegypti model. Figure S2: Response curves for important variables in the Aedes aegypti model. The curves show the mean response of the 10 replicate Maxent runs (red) and the mean ± one standard deviation (blue). (a) bio3. (b) bio19. (c) srad. (d) ndvi. Figure S3: Response curves for important variables in the Stomoxys calcitrans model. The curves show the mean response of the 10 replicate Maxent runs (red) and the mean ± one standard deviation (blue). (a) bio19. (b) bio6. (c) cattle. Figure S4. Habitat suitability map for Aedes aegypti. The warmer colors depict areas of high habitat suitability while cooler colors depict areas of low habitat suitability. Figure S5: Habitat suitability map for Stomoxys calcitrans. The warmer colors depict areas of high habitat suitability while cooler colors depict areas of low habitat suitability. Figure S6: Habitat suitability of LSD combined vector. The warmer colors depict areas of high habitat suitability while cooler colors depict areas of low habitat suitability. Table S1: Filtering of occurrence records. Table S2: Attributed values for the global LSD cases standard deviation ellipse, 2006–September 2022. [file 2349173.f1.docx]

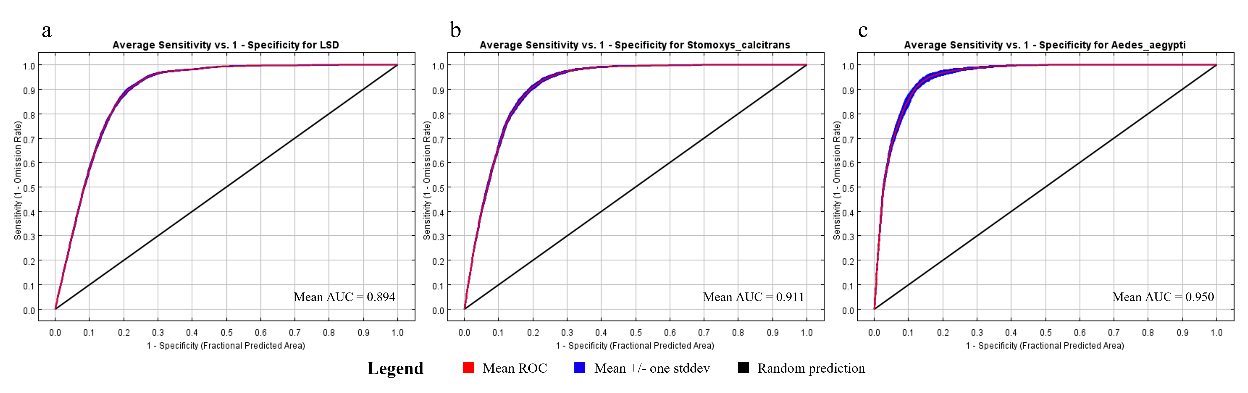
Figure S1. Results of the model evaluation based on the area under the receiver operating characteristic curve. The curves show the mean ROC of the 10 replicate Maxent runs (red) and the mean +/- one standard deviation (blue). The black line indicates random prediction. (a) LSD model. (b) *Stomoxys calcitrans* model. (c) *Aedes aegypti* model.


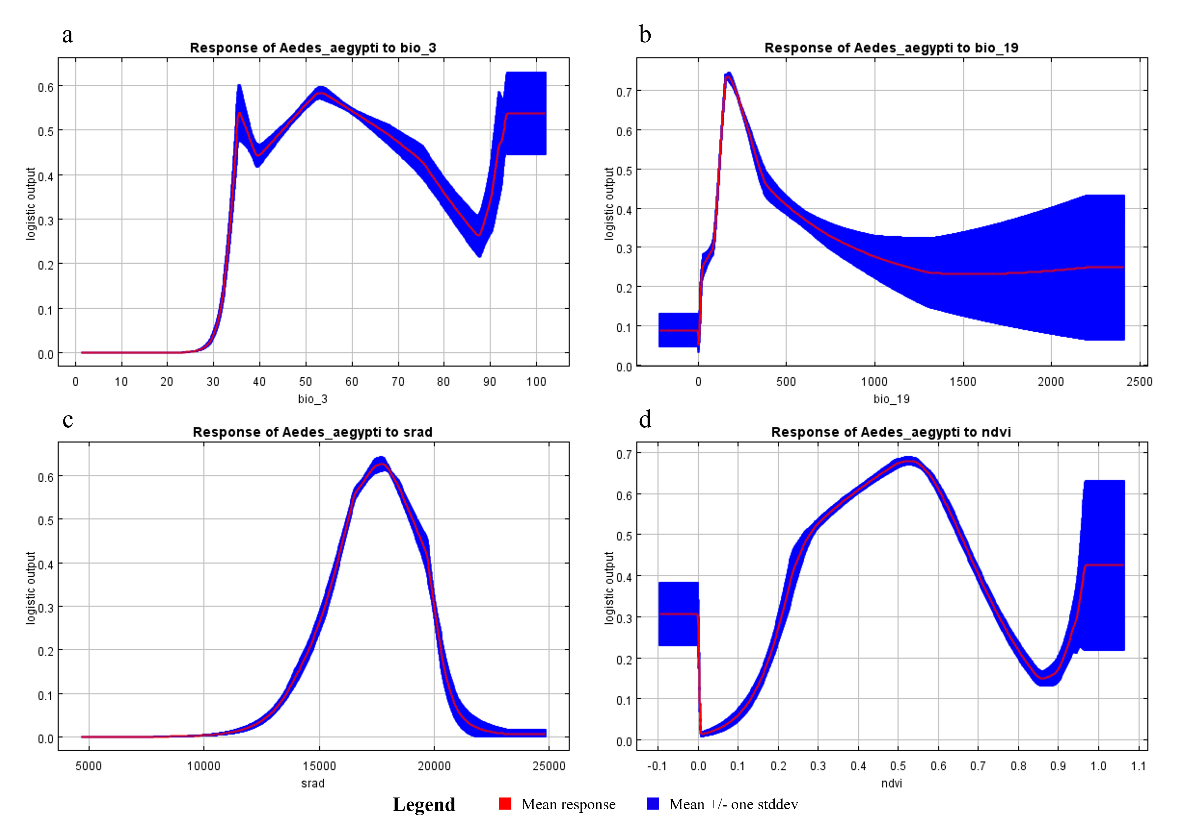


Figure S2. Response curves for important variables in the *Aedes aegypti* model. The curves show the mean response of the 10 replicate Maxent runs (red) and the mean +/- one standard deviation (blue).

(a) bio3. (b) bio19. (c) srad. (d) ndvi.


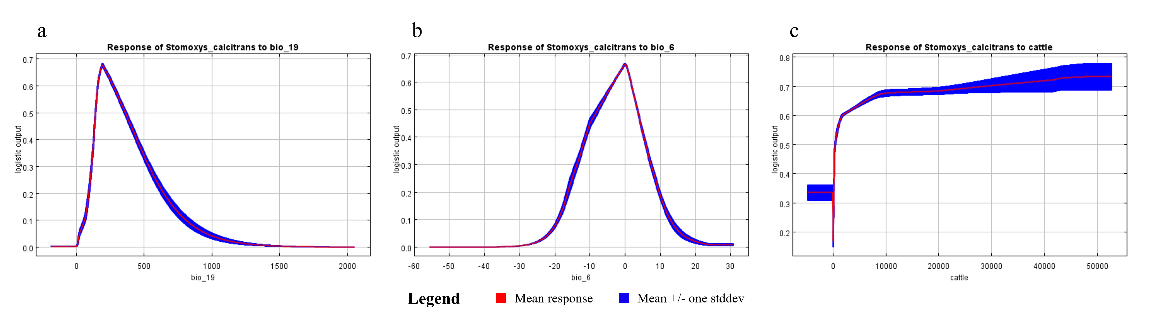


Figure S3. Response curves for important variables in the *Stomoxys calcitrans* model. The curves show the mean response of the 10 replicate Maxent runs (red) and the mean +/- one standard deviation (blue). (a) bio19. (b) bio6. (c) cattle.


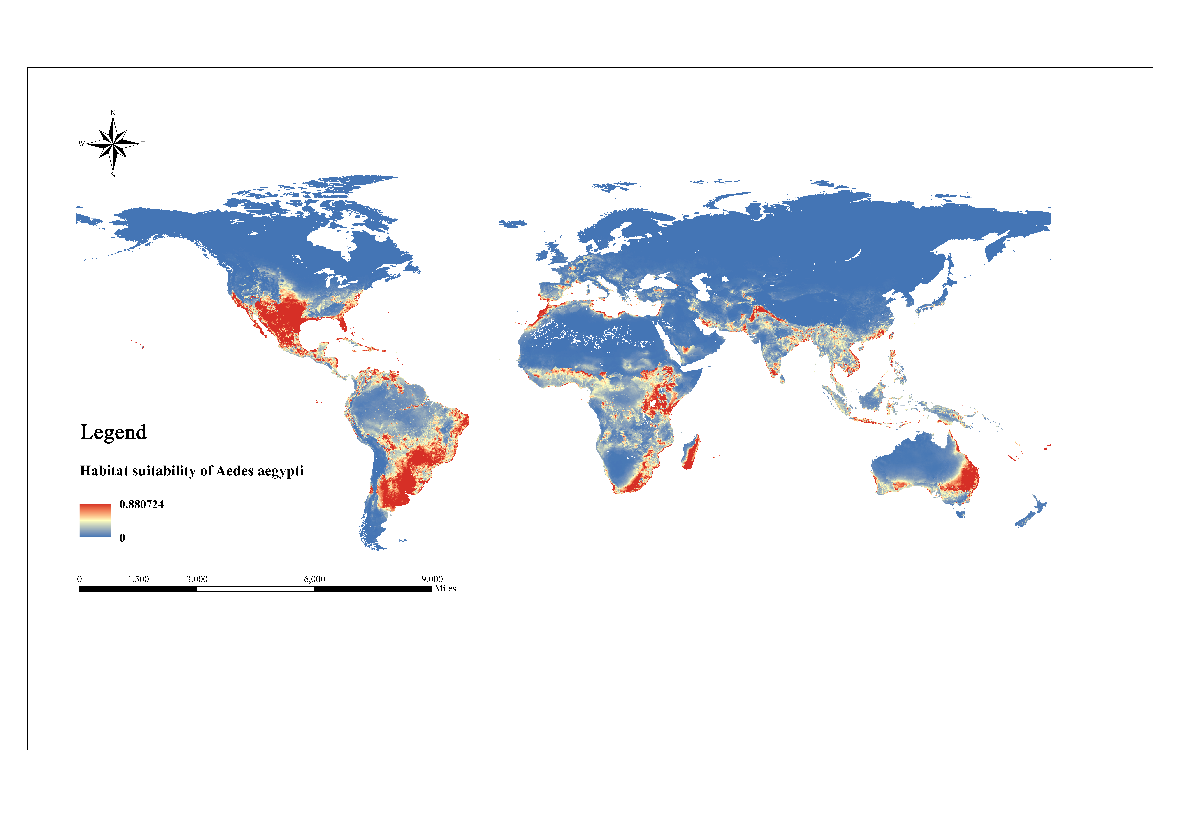


Figure S4. Habitat suitability map for *Aedes aegypti.* The warmer colors depict areas of high habitat suitability while cooler colors depict areas of low habitat suitability.


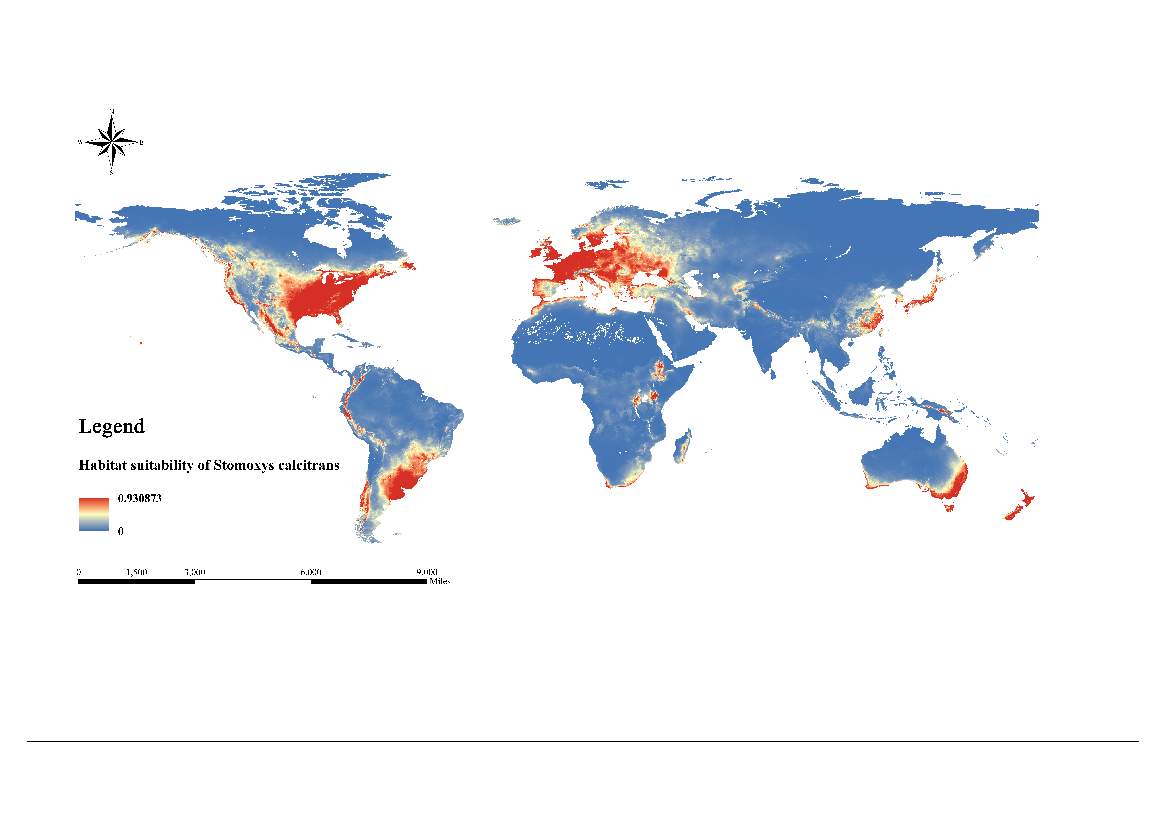
Figure S5. Habitat suitability map for *Stomoxys calcitrans.* The warmer colors depict areas of high habitat suitability while cooler colors depict areas of low habitat suitability.


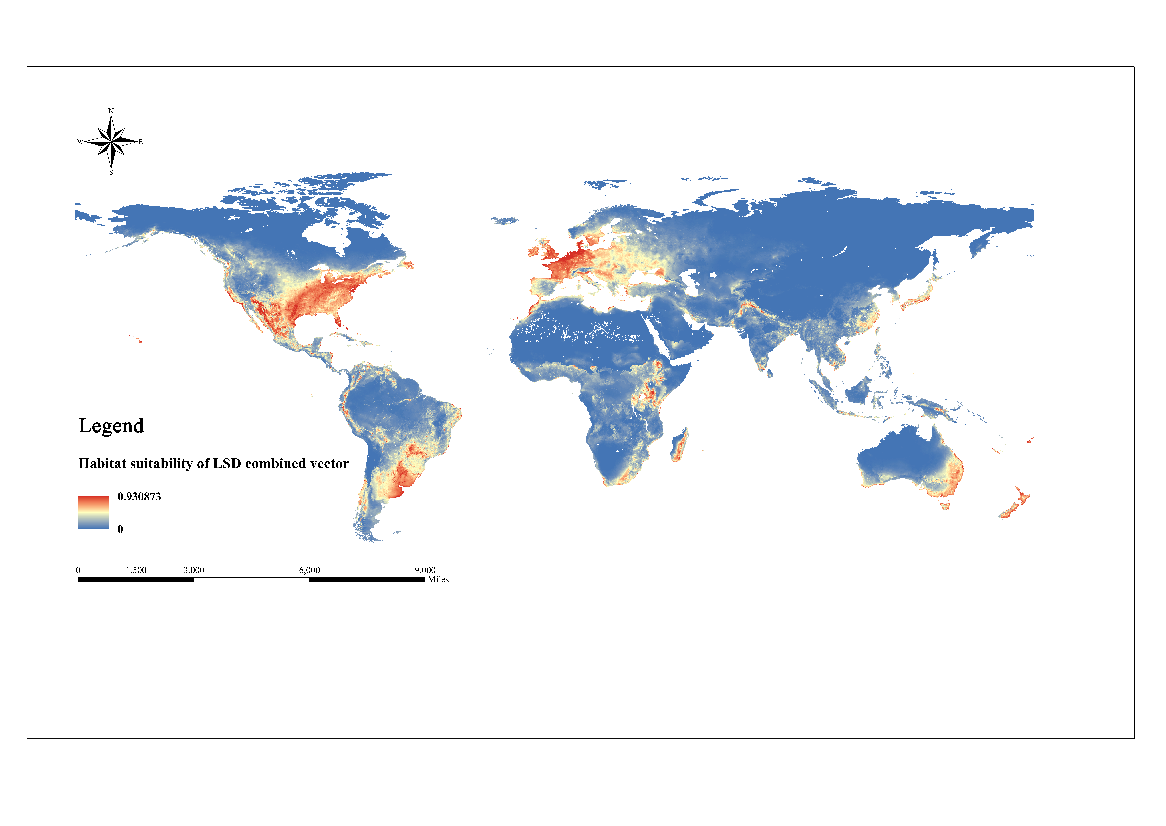


Figure S6. Habitat suitability of LSD combined vector. The warmer colors depict areas of high habitat suitability while cooler colors depict areas of low habitat suitability.

Table S1. Filtering of occurrence records

|  | Number of occurrences | Delete points | Final selected points |
| --- | --- | --- | --- |
| LSD | 4316 | 2120 | 2196 |
| *Aedes aegypti* | 13675 | 13065 | 610 |
| *Stomoxys calcitrans* | 2544 | 1059 | 1485 |

Table S2. Attributed values for the global LSD cases standard deviation ellipse, 2006- September 2022

| Time | Shape_Area | Center | XStdDist | YStdDist | Rotation |
| --- | --- | --- | --- | --- | --- |
| 2006-2009 | 2227.06 | 24.42, 17.43 | 25.86 | 27.41 | 6.09 |
| 2010-2013 | 101.66 | 34.78, 32.34 | 2.77 | 11.69 | 64.32 |
| 2014-2017 | 576.98 | 26.32, 40.12 | 12.34 | 14.89 | 35.32 |
| 2018-2022(9) | 1340.05 | 97.40, 20.03 | 25.84 | 16.51 | 120.50 |
